# Prospect of Fiduciary Duty as a Mitigating Factor for Healthcare Expenses

**DOI:** 10.1017/jme.2025.10219

**Published:** 2026

**Authors:** Dana Muir

**Affiliations:** University of Michigan Stephen M Ross School of Business, United States

**Keywords:** ERISA, employer-sponsored insurance, employer fiduciaries, employer health plans, group health plans

## Abstract

Employer complaints about rising health care costs have been ubiquitous for a number of years. Survey evidence, though, finds widespread employer shortcomings in the processes they use to select and monitor health plan service providers. The evidence provides a data-driven basis to assess whether employers meet their fiduciary obligations, leading to the conclusion that employers need to increase their diligence or face the possibility of significant liability. The increased diligence may also mitigate health care costs.

Employers complain about the rising costs of providing health care coverage to their employees. In their article “ERISA and the Failure of Employers to Perform Their Fiduciary Duties: Evidence from a Survey of Health Plan Administrators,” Richman et al. provide evidence from a survey of employers that a significant proportion of employers fail to take basic evaluative actions when choosing and monitoring plan service providers that could mitigate the cost of their health plans.^1^ The survey data provide a robust basis for the authors to assess the extent to which employers’ lack of diligence may violate their fiduciary duties.^2^

The article provides a succinct overview of the fiduciary obligations imposed by the Employee Retirement Income Security Act (ERISA) on employers that sponsor employee benefit plans, including health benefit plans.^3^ It then explains the results of a pioneering survey that assessed how employers choose providers to their health plans and judge the value of those plans.^4^ The results are stark. First, a significant percentage of employers are leaving money on the table, money that could reduce plan expenses and provide better health services to their employees. Second, those employers risk significant liability for violations of their fiduciary duties.

More than 565 legal actions have alleged excessively high fees in employer-sponsored retirement plans that result from the failure of employers to meet their fiduciary obligations.^5^ As Richman et al. observe, only recently has this type of litigation challenged high fees in health plans.^6^ Health plan cases are beginning to claim that employers have violated their fiduciary obligation of prudence through a lack of diligence in selecting and monitoring service providers, including pharmacy benefit managers (PBMs). The lack of diligence may result in higher fees that are passed along to employees through costs such as copays, the employees’ portion of health care premiums, or by decreases in other compensation components.^7^

Although both types of litigation allege excessive fees, health plan fee litigation encounters some challenges that do not exist in the retirement plan cases. Most notably, fees directly decrease the value of an individual’s retirement savings, so it is relatively simple to identify a specific injury to the saver. In contrast, employees confront difficulty in identifying a specific injury from high health plan fees. Even if the employees pay a portion of the plan premiums or have out-of-pocket costs, courts find that in the short term the employer bears the residual risk of excessive fees. Courts generally have dismissed the health care fee cases that have proceeded very far, deciding that employees have not shown the allegedly high fees caused them concrete harm (injury-in-fact).^8^ That does not mean that employers should not be concerned about liability for fiduciary breach. Litigants will continue to refine their claims as the legal principles in this area continue to develop.

The legal requirement that employees must show injury-in-fact to pursue a claim is in tension with economic logic. The survey conducted by Richman et al. finds that the overwhelming majority of employers fail to use standard approaches to reduce costs in their health plans.^9^ The authors observe that from an economic standpoint this is rational employer behavior. Because the market value of labor is fixed, higher health plan costs cause a reduction in salaries or benefits. Employers would not capture any of the increased costs spent on expanded diligence. The problem for employees in health care fee cases is to translate the economic theory to proof those fees caused an identifiable decrease in other compensation from the employer or in them bearing a larger portion of health plan costs.

In addition to a lack of employer effort in increasing the value of their health plans, the Richman et al. survey also found numerous other weaknesses in how employers engaged, or failed to engage, with their employees and health plan service providers. For example, few employers measured more than 10 of the possible 15 performance elements that could be used to evaluate health plans.^10^ Almost half (43%) measured fewer than 5 of the 15 elements.^11^ Even lower percentages of employers monitored their employees’ views about their health plan or the plan administrator (20%).^12^

As the authors recognize, employers face a complex task in meeting their obligation of prudence when selecting and monitoring health plan service providers.^13^ That task is becoming increasingly onerous. In 2024, the Department of Labor (DOL) clarified that its cybersecurity guidance applies to health plan service providers.^14^ Expanded information availability, such as mandatory posting of payer-specific pricing by hospitals, both enhances the amount of useful data and requires new methods of analysis.^15^ The difficulty inherent in assessing health plan service providers does not excuse employers from meeting the diligence obligations they owe their employees. Instead, it means employers must be more attentive to their plan obligations and hire consultants or other experts if they do not have the proficiency to do so on their own.

The Richman et al. survey results provide evidence that some employers may be violating their fiduciary duty of prudence in three ways. First, it appears that many do not meet the DOL’s guidance for information gathering and analysis when selecting service providers. Second, the Supreme Court has imposed a monitoring duty on sponsors of retirement plans, and the same statutory requirement applies to employers that sponsor health plans.^16^ Yet, as just noted, few employers broadly measure the performance of their plans or seek employee feedback about their plan’s value. Third, in light of their failure to engage in diligent selection and monitoring of service providers, it seemingly is impossible for employers to show that their plans are not paying more than reasonable compensation to those providers.

This article’s important contributions are of interest to health plan sponsors, those interested in health policy, and employee advocates. It is an implicit indictment of the US health care system, in which employers act as intermediaries between covered employees and health care service providers but do not have a direct economic incentive to minimize plan costs. In response, it offers important insights on how and why employers need to be more attentive to their fiduciary obligations when selecting and monitoring health plan service providers. The potential for liability provides a possible corrective for the market inefficiencies.
